# Integration of Transcriptomics and Proteomics Analysis Reveals the Molecular Mechanism of *Eriocheir sinensis* Gills Exposed to Heat Stress

**DOI:** 10.3390/antiox12122020

**Published:** 2023-11-21

**Authors:** Chenchen Shen, Guangpeng Feng, Feng Zhao, Xiaorong Huang, Min Wang, Haihua Wang

**Affiliations:** 1 East China Sea Fisheries Research Institute, Chinese Academy of Fishery Sciences, Shanghai 200090, China; shencc7@163.com (C.S.); zhaof@ecsf.ac.cn (F.Z.); hxr828@126.com (X.H.); queenwang916@163.com (M.W.); 2 College of Fisheries and Life sciences, Shanghai Ocean University, Shanghai 200090, China; 3 Jiangxi Institute for Fisheries Sciences, Poyang Lake Fisheries Research Centre of Jiangxi, Nanchang 330039, China; jxswhh@163.com

**Keywords:** Chinese mitten crab, gills, high temperature, multi-omics, antioxidation mechanism

## Abstract

Heat stress is an increasingly concerning topic under global warming. Heat stress can induce organisms to produce excess reactive oxygen species, which will lead to cell damage and destroy the antioxidant defense of aquatic animals. Chinese mitten crab, *Eriocheir sinensis*, is sensitive to the change in water temperature, and parent crabs are more vulnerable during the breeding stage. In the present study, the multi-omics responses of parent *E. sinensis* gills to heat stress (24 h) were determined via transcriptome and proteome. The integrative analysis revealed that heat shock protein 70 (HSP70) and glutathione s-transferase (GST) were significantly up-regulated at gene and protein levels after heat stress, indicating that HSP70 and the antioxidant system participated in the regulatory mechanism of heat stress to resist oxidative damage. Moreover, the “Relaxin signaling pathway” was also activated at gene and protein levels under 30 °C stress, which implied that relaxin may be essential and responsible for reducing the oxidative damage of gills caused by extreme heat stress. These findings provided an understanding of the regulation mechanism in *E. sinensis* under heat stress at gene and protein levels. The mining of key functional genes, proteins, and pathways can also provide a basis for the cultivation of new varieties resistant to oxidative stress.

## 1. Introduction

Extreme heat weather occurs frequently, and water temperature increases obviously under the influence of global warming [1]. Heat stress can elevate the production of reactive oxygen species (ROS) in aquatic animals, and excess ROS will lead to cell damage and destroy the antioxidant defense, which negatively affects the growth and development of organisms [2,3,4]. To reduce the oxidative stress caused by high temperature, various antioxidants, such as superoxide dismutase (SOD), glutathione s-transferase (GST), and glutathione peroxidase (GSH-Px), function jointly to keep redox balance [5]. Moreover, heat shock proteins (HSPs), especially HSP70, were found to counteract extreme high-temperature environment and minimize cell damage caused by high ROS levels [6,7]. Thus, antioxidants and heat shock proteins play an important role in resistance to excess ROS under heat stress. The response of animals to heat stress consists of a highly ordered series of events, mainly including the rapid changes in gene expression and the synthesis of proteins involved in environmental adaptation [8]. Due to the robustness and reliability of identifying gene expression patterns and functions, the comparative transcriptome has been widely used to analyze the molecular mechanisms in aquatic animals exposed to heat stress [9,10,11]. Additionally, proteome can comprehensively investigate the changes in proteins and signaling pathways within tissues under heat stress [12]. Therefore, the integrative analysis of the transcriptome and proteome has become a research hotspot in the response of complex molecules to heat stress [13,14,15].

*Eriocheir sinensis*, a representative crab in crustaceans, is a crucial economic and ecological aquatic species in China, and is also an invasive species in Europe and America [16,17]. *E. sinensis* belongs to an aquatic ectotherm which is sensitive to the change in water temperature, and parent crabs are more vulnerable during the reproduction period [18]. Water temperature can affect the normal behavior of *E. sinensis* such as predation, copulation, and spawn. Previous reports have found that the appropriate temperatures for juvenile, adult, and parent *E. sinensis* were 20–25 °C, 22–30 °C, and 8–11 °C, respectively [19,20,21]. Meanwhile, studies of acute heat stress have shown that high temperature can affect antioxidant activity, immune defense, and metabolic ability, leading to the disorder of free radical metabolism [22,23]. However, most studies on the heat stress of *E. sinensis* focused on the juvenile crabs and the physiological–biochemical level, which cannot reveal the response mechanism of high temperature on *E. sinensis*.

The gills, a vital organ participating in respiratory, metabolism, and stress response, are in direct contact with water and are continuously exposed to complex water environments [24,25,26]. The gills are easily damaged by environmental factors, such as salinity, ammonia nitrogen, and heavy metal [27,28,29]. Moreover, gills are often used as a target organ under heat stress for transcriptomic or proteomic studies [30,31,32]. The gills contain abundant innate immune cells and stress pathways against external stress [33,34]. Therefore, the gills are an appropriate object to study the molecular mechanism of heat stress in this study.

In order to understand the molecular mechanisms in gills’ response to the heat stress of *E. sinensis*, the transcriptome and proteome were collectively performed via *Illumina* sequencing technology and label-free quantitative proteomics technology. The genes and proteins related to antioxidation have been focused on closely. Our results can provide new insight into the regulatory events under heat stress at transcriptional and translational levels, and improve the understanding of the antioxidation mechanism of *E. sinensis* under heat stress.

## 2. Materials and Methods

### 2.1. Animal and Acute Heat Stress Treatment

A total of 120 parent *Eriocheir sinensis* (adult male-to-female ratio 1:1) were collected from the Shanghai Hubao Aquaculture Cooperatives (Baoshan District, Shanghai, China) in November 2022. The average weights of female and male crabs were 122.00 ± 12.25 g and 144.25 ± 11.32 g, respectively. To prevent mating, crabs were temporarily raised in two tanks according to sex, and gradually domesticated from 18 ± 0.5 °C (the indoor water temperature) to 10 ± 0.5 °C (the initial experimental temperature), which is an appropriate temperature for parent crabs [12], with a decrease of 1 °C/d. Then, crabs were acclimatized at 10 ± 0.5 °C for three weeks. During domestication, crabs were fed with puffed compound fodder (Changzhou Haida Biological Feed Co., Ltd., Changzhou, China) every night, and the main ingredients were fish meal, soybean meal, rapeseed meal, and so on. The quality parameters of water were monitored to maintain the temperature 10 ± 0.5 °C, pH of 7.0–7.5, and dissolved oxygen > 6 mg/L.

According to our previous research, parent crabs have a better ability to adapt to high temperature, and the gills showed slight pathological changes at 20 °C and obvious pathological changes at 30 °C. Thus, 20 °C and 30 °C were chosen as experimental temperatures. In the formal experiment, parent crabs were randomly divided into three groups: medium-temperature (MT), high-temperature (HT), and control (CT) groups. Each group contained 30 crabs (adult male-to-female ratio 1:1), and no food was provided in the formal period. The CT group was constantly cultured at 10 ± 0.5 °C. The water temperature of MT group was increased from 10 °C to 20 °C, and the water temperature of HT group was increased from 10 °C to 30 °C, altering the water temperature in the tank at a constant rate of 3 °C per 1 h (the optimum heating rate obtained by pre-experiment). A 2000 W heating rod and temperature controller (temperature control range 0–80 °C, temperature sensitivity 0.1 °C, Korea A-MI) were equipped to adjust water temperature. After reaching the target temperature, six experimental crabs (3 female crabs and 3 male crabs) in each group (MT, HT, and CT) were quickly removed at 24 h for gills dissection. The samples were pooled (sextuple repetition) and immediately flash-frozen in liquid nitrogen for further analysis.

### 2.2. Transcriptome Sequencing and Analysis

The total RNA was extracted using TRIzol reagent (Aidlab, Beijing, China) following the manufacturer’s procedure. The quality and integrity of total RNA were analyzed via NanoDrop 2000 spectrophotometer (NanoDrop Technologies, Wilmington, DE, USA). The cDNA libraries construction, transcriptome sequencing, and data analysis were accomplished by using Shanghai Majorbio Bio-pharm Technology Co., Ltd. (Shanghai, China) on the *Illumina* NovaSeq 6000 platform. To obtain high-quality clean data, raw data were initially filtered via TransRate (http://hibberdlab.com/transrate/ (accessed on 20 December 2022)) and CD-HIT (http://weizhongli-lab.org/cd-hit/ (accessed on 20 December 2022)). The assembly integrity of transcriptome was evaluated using BUSCO (http://busco.ezlab.org (accessed on 20 December 2022)). The transcriptome de novo assembly was performed using Trinity. Then, the unigenes were matched with sequences in BLAST against the Non-redundant (NR), STRING, SWISS-PROT, Protein family (Pfam), EGGNOG, Gene ontology (GO), and the Kyoto Encyclopedia of Genes and Genome (KEGG) databases, with a cut-off E-value of 1 × 10^−5^. RSEM normalization (http://deweylab.biostat.wisc.edu/rsem/ (accessed on 21 December 2022)) was used to perform expression levels for mRNA by calculating fragments per kilobase of exon per million mapped fragments (FPKM). The differentially expressed genes (DEGs) were selected with a fold change ≥ 2 and *p*-value < 0.01 via DEGseq. GO and KEGG functional enrichment analysis were conducted using Goatools (https://github.com/tanghaibao/GOatools (accessed on 22 December 2022)) and KOBAS (http://kobas.cbi.pku.edu.cn/home.do (accessed on 22 December 2022)), respectively. The corrected *p*-value < 0.05 was defined as significantly enriched terms or pathways.

### 2.3. Proteome Sequencing and Analysis

Proteome sequencing and data analysis were accomplished via label-free quantitative proteomics technology. The proteins of gills were successively extracted and quality-controlled through bicinchoninic acid assay and sodium dodecyl sulfate polyacrylamide gel electrophoresis. Subsequently, proteins were enzymatically hydrolyzed into peptides and LC-MS/MS (liquid chromatography tandem mass spectrometry) was applied. Based on Proteome Discoverer TM Software 2.2, the proteins were identified from raw data against the NR, SWISS-PROT, Pfam, Subcell-location, GO, and KEGG databases. According to the criteria of fold change ≥ 1.2 and *p*-value < 0.05, the differentially expressed proteins (DEPs) were identified for MT, HT, and CT groups. All DEPs were subjected to GO and KEGG enrichment analyses.

### 2.4. Screening of Genes and Proteins Related to Antioxidation

The keyword screening method was used to screen antioxidase and heat shock family in all differentially expressed gene and protein databases. The antioxidase included superoxide dismutase (SOD), catalase (CAT), glutathione (GSH), glutathione peroxidase (GSH-Px), glutathione S-transferase (GST), and peroxiredoxin. The members of heat shock family mainly included heat shock proteins 70 (HSP70), heat shock proteins 60 (HSP60), and heat shock proteins 90 (HSP90).

### 2.5. Quantitative Real-Time Polymerase Chain Reaction (qRT-PCR)

Eight genes, including phosphoinositide-3 kinase (*PIK3CA*), caspase-7 (*CASP7*), heat shock protein 70 (*HSP70*), heat shock protein 90 (*HSP90*), eukaryotic translation initiation factor 6 (*EIF6*), ATP synthase (*ATPase*), v-type proton ATPase (*V-ATPase*), and anti-lipopolysaccharide factor (*ALF*) were selected and analyzed via quantitative real-time polymerase chain reaction (qRT-PCR). *β-actin* was selected as the endogenous control gene in this study. The gene-specific primers were designed using Primer Premier 5.0 (Table 1). All genes were submitted to GenBank (SUB13970835), and accession numbers were in Table S1. The conditions of PCR amplification were set as: 95 °C for 5 min, then 35 cycles of 95 °C for 30 s, 55 °C for 30 s, and 72 °C for 1 min. The qRT-PCR analysis was performed on an Applied Biosystem 7500 real-time PCR system (Applied Biosystems, Thermo Fisher Scientific, Waltham, MA, USA) with the 2 × SYBR Green qPCR Mix (Aidlab Biotechnologies Co., Ltd., Beijing, China), while the relative levels of gene expression among the different samples were measured using the 2^−ΔΔCt^ method [35].

## 3. Results

### 3.1. Overall Statistics for Transcriptomic and Proteomic Sequencing

**Figure 1:**
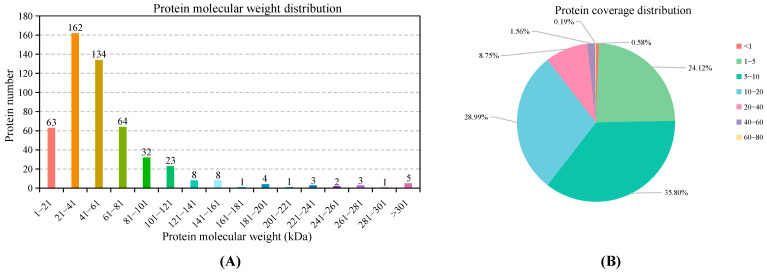
Protein molecular weight distribution (**A**) and protein coverage distribution (**B**).

The transcriptomic of gills under heat stress was carried out via *Illumina* paired-end sequencing technology, which generated 150,986,054 raw reads. After filtering low-quality reads, 49,537,350, 51,705,776 and 48,425,930 clean reads were obtained in the CT, MT, and HT groups with a GC content of 44.92%, 45.01%, and 49.16%, respectively. The de novo transcriptome assembly yielded 77,708 transcripts and 49,754 unigenes with an average length of 920.74 bp, an N50 of 1740 bp, and a GC content of 46.47% (Table S2). The statistical summary of gene functional annotations via BLAST analysis is shown in Table S3. The proteome of gills matched 5008 spectrogram and identified 750 proteins. After grouping, the number of confirmed proteins was 514. The relative molecular masses of the identified proteins were mostly distributed in the range of 21–61 kDa, and 24.70% of protein sequence coverage was less than 5% (Figure 1). The statistical summary of protein functional annotations via BLAST analysis is shown in Table S4.

### 3.2. Comparison and Enrichment Analysis of DEGs

**Figure 2:**
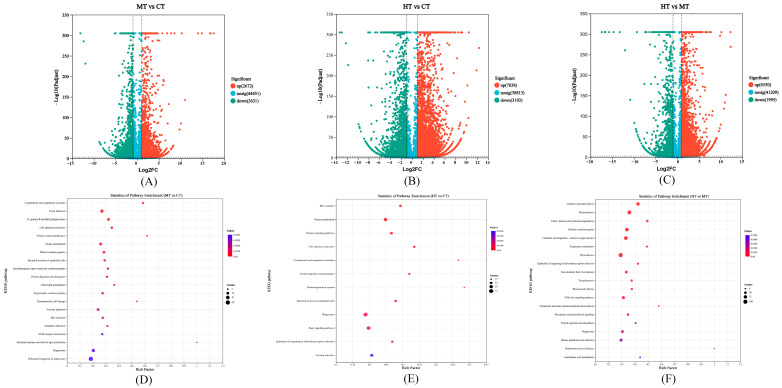
Volcano plots for DEGs in the MT vs. CT (**A**), HT vs. CT (**B**), and HT vs. MT (**C**); and KEGG enrichment analysis of DEGs in the MT vs. CT (**D**), HT vs. CT (**E**), and HT vs. MT (**F**).

Abundant DEGs were identified in gills transcriptome, including 5303 DEGs with 2672 up-regulated and 2631 down-regulated genes in MT vs. CT; 10,941 DEGs with 7838 up-regulated and 3103 down-regulated genes in HT vs. CT; and 8545 DEGs with 6550 up-regulated and 1995 down-regulated genes between HT vs. MT (Figure 2A–C). The number of up-regulated DEGs in HT vs. CT was 2.93 times higher than that in MT vs. CT, and more DEGs (76.65%) were significantly up-regulated in HT vs. MT, indicating that 30 °C heat stress can induce more genes in the gills of *E. sinensis*. Meanwhile, DEGs related to the heat shock protein family, antioxidant system, energy metabolism, and immune defense were significantly differentially expressed (Table 2). KEGG enrichment analysis indicated that 25 and 13 pathways were significantly enriched in MT vs. CT and HT vs. CT, respectively (Figure 2D,E), including nine co-enrichment KEGG pathways, such as “Complement and coagulation cascades”, “Phagosome”, and “Protein digestion and absorption” (Figure S1). Clotting factor B, clotting factor G, and prophenoloxidase-activating enzyme (*PPAE*) were up-regulated in the “Complement and coagulation cascades”. C-type lectin (*CTL*), integrin, and ras-related C3 botulinum toxin substrate 1 (*RAC1*) were up-regulated, but cathepsin L (*CTSL*) and v-type proton ATPase (*V-ATPase*) were down-regulated in the “Phagosome”. Notably, more DEGs were up-regulated in MT vs. CT in the “Phagosome” than HT vs. CT. The specific enrichment pathways of MT vs. CT mainly included “Fc gamma R-mediated phagocytosis”, “Regulation of actin cytoskeleton”, and “Toll and Imd signaling pathway”, and the specific enrichment pathways of HT vs. CT mainly included “Relaxin signaling pathway” and “Renin–angiotensin system” (Table 3, Figure S2). Furthermore, the top two enriched pathways in HT vs. MT were “Oxidative phosphorylation” and “Thermogenesis” (Figure 2F and Figure S3), and abundant DEGs were down-regulated, including *ATPase*, V-ATPase, *SDH*, *ND*, *COX*, *CYC*, and so on.

### 3.3. Comparison and Enrichment Analysis of DEPs

**Figure 3:**
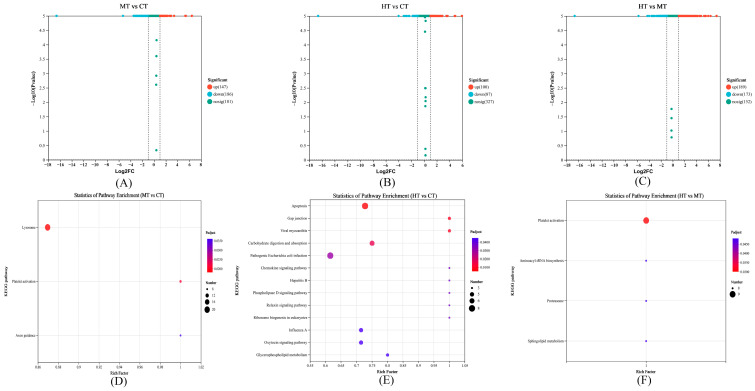
Volcano plots for DEPs in the MT vs. CT (**A**), HT vs. CT (**B**), and HT vs. MT (**C**); and KEGG enrichment analysis of DEPs in the MT vs. CT (**D**), HT vs. CT (**E**), and HT vs. MT (**F**).

Abundant DEPs were identified in gills proteome, including 333 DEPs with 147 up-regulated and 186 down-regulated genes in MT vs. CT, 187 DEPs with 100 up-regulated and 87 down-regulated genes in HT vs. CT, and 362 DEPs with 189 up-regulated and 173 down-regulated genes in HT vs. MT (Figure 3A–C). The number of up-regulated DEPs in MT vs. CT was slightly higher than that in HT vs. CT, indicating that 20 °C stress can induce more proteins in the gills of *E. sinensis*. Meanwhile, DEPs related to the heat shock protein family, antioxidant system, and energy metabolism were also significantly differentially expressed (Table 4). KEGG enrichment analysis indicated that three and 13 pathways were significantly enriched in MT vs. CT and HT vs. CT, respectively (Figure 3D,E). For MT vs. CT, scavenger receptor class B-I (SR-BI) and v-ATPase were up-regulated, and the cathepsin family (CTSL, CTSA, CTSC), arylsulfatase, and β-glucuronidase were down-regulated in the “Lysosome”. MAP kinase (MAPK), talin-2, actin, and myosin regulatory light chain 2 were down-regulated in the “Platelet activation” (Figure S4). For HT vs. CT, MAPK and cathepsin family (CTSL and CTSC) were up-regulated in “Apoptosis”; MAPK and β arrestin-1 were up-regulated in “Relaxin signaling pathway”; phospholipase D delta was down-regulated in “Phospholipase D signaling pathway” (Figure S5).

### 3.4. Integrative Analysis of the Transcriptome and Proteome

**Figure 4:**
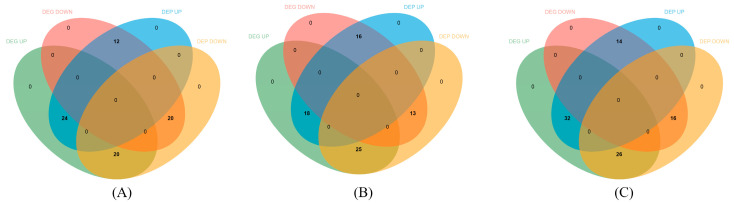
Differentially expressed genes and proteins association analysis in the MT vs. CT (**A**), HT vs. CT (**B**), and HT vs. MT (**C**).

A total of 490 proteins and genes were associated, including 76 associated differentially expressed genes and proteins (DEGs/DEPs) with 24 up-regulated co-expressed genes and proteins (co-up-DEGs-DEPs) and 20 down-regulated co-expressed genes and proteins (co-down-DEGs-DEPs) in MT vs. CT; 72 associated DEGs/DEPs with 18 co-up-DEGs-DEPs and 13 co-down-DEGs-DEPs in HT vs. CT; and 88 associated DEGs/DEPs with 32 co-up-DEGs-DEPs and 16 co-down-DEGs-DEPs in HT vs. MT (Figure 4). HSP70, GST, integrin β, chitinase 1, and pseudo-hemocyanin in MT vs. CT, and hemocyanin, ficolin-1, and retinol dehydrogenase 12 in HT vs. CT were up-regulated (Table S5). Moreover, eukaryotic translation initiation factor 6 (EIF6), spliceosome RNA helicase, and ras-related GTP binding protein 7 (Rab7) were all down-regulated in MT vs. CT and HT vs. CT.

**Figure 5:**
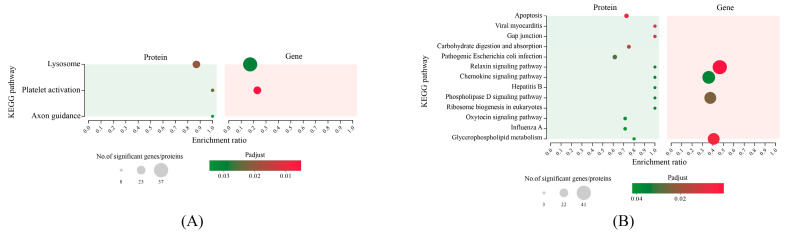
KEGG enrichment of differentially expressed genes and proteins’ association analysis in the MT vs. CT (**A**) and HT vs. CT (**B**).

From the 490 associated DEGs-DEPs, a total of 236 associated DEGs-DEPs were analyzed for the enrichment of the KEGG metabolic pathway (Figure 5). The “Lysosome” and “Platelet activation” were significantly enriched in MT vs. CT. In “Lysosome”, SR-BI and v-ATPase were up-regulated, and the cathepsin family (CTSL, CTSA, CTSC), arylsulfatase, and β-glucuronidase were down-regulated. In “Platelet activation”, more up-regulated DEGs were significantly enriched, including glycoprotein IIb/IIIa (GPIIb/IIIa), tyrosine-protein kinase Src (cSrc), actin, and talin-2, and then DEPs of “Platelet activation” were up-regulated in HT vs. MT, including MAPK, talin-2, myosin regulatory light chain, and actin (Figure S6). “Relaxin signaling pathway” and “Phospholipase D signaling pathway” were significantly enriched in HT vs. CT.

**Figure 6:**
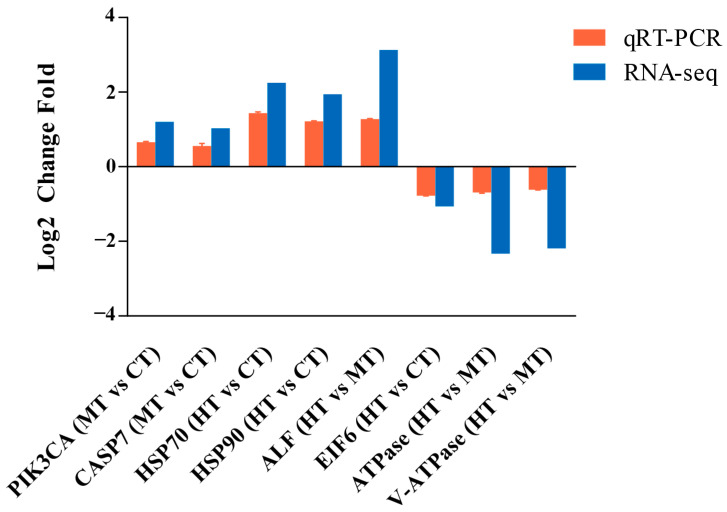
Comparison of the expression profiles of eight genes as determined via RNA-seq and validated through qRT-PCR in the gills of *Eriocheir sinensis*.

In order to verify the sequencing results, eight genes were selected randomly for further confirmation via qRT-PCR. The results confirmed a good consistency of the qRT-PCR and RNA-Seq data (Figure 6).

## 4. Discussion

### 4.1. The Differences of DEPs and DEGs in the Integrative Analysis

In this study, 9462 DEGs and 427 DEPs were identified at the gene and protein levels, respectively, and from them, 123 DEGs-DEPs were co-expressed. The number of DEGs was significantly higher than that of DEPs, which is a common situation in the integrative analysis of transcriptomics and proteomics [25,36]. Alli Shaik and Lundberg both found that the protein expression level mainly depends on its mRNA level when an organism is in homeostasis chronically, but the translational level will change differently from the transcriptional level if the homeostasis is broken [37,38]. Then, due to a series of complex regulatory mechanisms such as post-transcriptional modification, including phosphorylation and ubiquitylation, the genes and proteins are not expressed synchronously at the transcriptional and translational levels [39,40,41]. Moreover, some proteins may be masked by high-abundance proteins, restricting their detection, and the mRNAs could not be efficiently translated, resulting in low or null protein levels. Additionally, the normal protein synthesis will be inhibited if the individual is subjected to heat stress [42], and this may also be the cause of the low amount of protein expression. Therefore, heat stress may disrupt the cell state of gills (homeostasis) and inhibit the expression of protein. The post-transcriptional regulatory mechanisms may exist in the response to heat stress, which needs further study.

### 4.2. Co-Expression of HSP70 and GST at Gene and Protein Levels under Heat Stress

**Figure 7:**
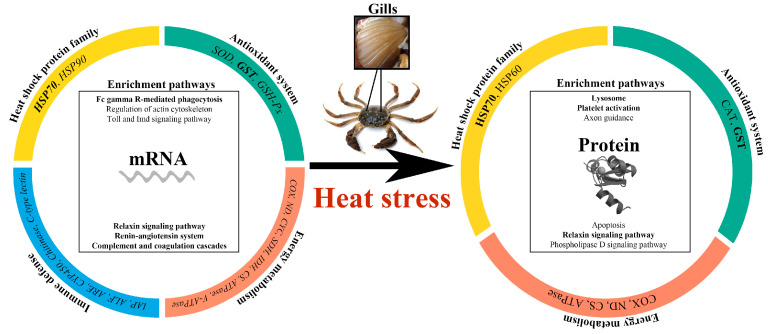
The regulation mechanisms of *Eriocheir sinensis* gill exposed to heat stress at mRNA and protein levels.

The co-DEG-DEPs with consistent expression patterns at the transcriptional and translational levels were further investigated, which may play a key role in response to heat stress (Figure 7). HSP70 is a member of the heat shock protein family, which can prevent protein folding, refold denatured protein, and degrade denatured protein [43]. Moreover, it has been demonstrated that HSP70 plays an important role in combating heat stress, and the highly expressed HSP70 gene can improve the heat tolerance of cells, thus inhibiting the generation of oxygen radical and the apoptosis rate induced by heat stress [6,44,45]. Previous studies have reported that HSP70 gene was significantly up-regulated in *Rimicaris exoculata*, *Penaeus monodon*, *Scylla paramamosain*, and *Litopenaeus vannamei* under heat stress (28–35 °C) [46,47,48]. In this study, HSP70 was up-regulated at both the gene and protein levels under heat stress, and the expression of HSP70 was significantly higher at 30 °C than 20 °C in this study, indicating that the highly expressed HSP70 is involved in the regulatory mechanism of heat stress, especially higher temperature. In addition to the heat shock protein family, the antioxidant system is also an important component of resistance to oxidative damage caused by heat stress [49]. Various antioxidants, including SOD, GSH-Px, and CAT, were significantly up-regulated at the gene or protein level, but only GST was co-up-regulated at two levels in this study. GST can effectively remove reactive oxygen species in time [50], thus the significant up-regulation of GST implied that the antioxidant system is activated in response to heat stress. Moreover, hemocyanin and pseudo-hemocyanin were significantly up-regulated at the gene and protein levels, which both belong to the arthropod hemocyanin superfamily [51]. The main function of hemocyanin is oxygen transport, and pseudo-hemocyanin functions as protein storage [52]. It is speculated that the hemocyanin superfamily may participate in the regulatory mechanism of gills under heat stress. Additionally, although the expression of genes and proteins related to energy metabolism and immune defense lacks a good consistency, the regulation of many energy metabolism enzymes and immune factors at the gene level still indicated that heat stress would impair energy metabolism and immune defense.

### 4.3. Effective Pathways Involved in Response to Heat Stress

In this study, ACE gene was up-regulated in the “Renin–angiotensin system”. ACE can catalyze the conversion of angiotensin I to angiotensin II, thus playing a role in constricting blood vessels and increasing blood pressure [53]. The up-regulation of the ACE gene can activate the renin–angiotensin system, which can increase platelet sensitivity and blood pressure, and promote thrombosis [54]. Notably, more up-regulated genes in MT vs. CT were significantly enriched in the “Platelet activation”, and more DEPs of “Platelet activation” were up-regulated in HT vs. MT, suggesting that platelet activation was enabled at both the transcriptional and translational levels with increasing temperature. Platelet activation refers to a series of reactions that occur in platelets during the early stage of hemostasis, which is also the key to thrombosis [55]. Glycoprotein IIb/IIIa (GPIIb/IIIa), a receptor-mediating platelet binding to fibrinogen, was up-regulated in MT vs. CT, which plays a key role in platelet activation [56]. GPIIb/IIIa can directly affect the downstream pathway “Complement and coagulation cascades”. The “Complement and coagulation cascades” was significantly enriched in MT vs. CT and HT vs. CT, and clotting factor B, clotting factor G, and PPAE genes were up-regulated. Clotting factors (B and G) belong to the serine protease family, and participate in the clotting system of crustaceans. When the tissue is damaged or infected by foreign pathogens, crustaceans can produce blood clots through the cascade reaction of the clotting system to prevent excessive bleeding and pathogen invasion [57]. Similarly, “Complement and coagulation cascades” was also significantly enriched, and the related genes in the gills were induced in *Oncorhynchus mykiss* under heat stress [58,59]. Moreover, the coagulation pathway-associated genes (coagulation factor II, fibrinogen gamma chain, and carboxypeptidase B2) were also up-regulated, and angiotensinogen was significantly up-regulated at the transcription and protein levels in *O. mykiss* under heat stress [60]. In general, the complement-mediated hemolysis, platelet activation, and thrombosis occur in humans under oxidative stress [61]. Therefore, acute heat stress may cause oxidative stress, which can induce gill damage, blood coagulation, and thrombosis in *E. sinensis*, resulting in increased blood pressure and physiological imbalance.

Two important innate immune defense pathways (“Lysosome” and “Phagosome”) were enriched. In MT vs. CT, “Lysosome” was significantly enriched at both gene and protein levels, and many cathepsin (CTSL, CTSA and CTSC) and β-glucuronidase were significantly down-regulated. Similarly, it was also found that the cathepsin family (CTSL, CTSC, CTSD), glucosylceramidase, lysosomal alphaglucosidase, and arylsulfatase B were down-regulated in *Procambarus clarkii* under heat stress [62]. These genes or proteins involved in the dissolution and digestion of foreign macromolecules in lysosomes were significantly down-regulated, implying that heat stress may affect the function and integrity of lysosomes [63]. Additionally, SR-BI was significantly up-regulated at both transcription and protein levels in the “Lysosome”. SR-BI participates in the macrophage migration and inflammation. After bacterial stimulation, SR-BI gene in *E. sinensis*, *Marsupenaeus japonicus* and *Litopenaeus vannamei* was up-regulated, indicating that SR-BI can enhance the phagocytosis of invading bacteria, thereby protecting shrimp and crabs from bacterial damage [64,65,66]. Therefore, the SR-BI gene may have the same function under heat stress. Notably, “Phagosome” was significantly enriched at the gene level under both 20 °C and 30 °C heat stress. Phagosome plays an important role in invertebrate resistance to bacterial infection [67]. In the “Phagosome”, CTL, integrin, and RAC1 were up-regulated, suggesting that the phagosome of *E. sinensis* was an important immune way in early response to heat stress. Meanwhile, “Fc gamma R-mediated phagocytosis” was significantly enriched at the gene level under 30 °C heat stress, also suggesting that phagocytosis plays a key immune function in response to heat stress.

Notably, most pathways related to stress were enriched in the MT group. It is speculated that this may be because a 30 °C stress severely damaged the tissue structure of gills, leading to branchial dysfunction and more missed pathways. The enriched pathways in the HT and MT groups indicated that the mechanisms of parent crabs coping with different heat stresses were discernible. Moreover, parent crabs may be more adaptable to 20 °C stress than 30 °C stress. Fortunately, based on the integrated proteomics and transcriptomic analysis, the “Relaxin signaling pathway” was significantly enriched at both the gene and protein levels under 30 °C stress (Figure 7). More DEGs were up-regulated in the “Relaxin signaling pathway”. Relaxin (RLN) is a polypeptide hormone, which can reduce the accumulation of peroxide products, inhibit oxidative damage, and improve the antioxidant capacity of endothelial cells [68,69]. RXFP1, the homologous receptor of RLN1 and RLN2 [70], and genes associated with cAMP production such as PI3K, AC, PKC, and CREB were up-regulated, promoting the generation of cAMP and thus activating gene transcription such as NOS1. Moreover, RLN2 has been proven to reduce cellular oxidative damage in hypoxic rats [71], but RLN is rarely reported in decapoda under heat stress. Therefore, although the “Relaxin signaling pathway” did not involve many DEPs at the protein level, the activation of “Relaxin signaling pathway” at the gene level still implied that RLN may play an important role in the response of *E. sinensis* gills to extreme heat stress.

## 5. Conclusions

In summary, we first performed an integrated proteomics and transcriptomic analysis of the response of *E. sinensis* gills to heat stress. Several candidate genes and proteins, especially HSP70 and GST with high consistency in both gene and protein levels, were involved in resisting heat stress. Moreover, functional analysis showed that the “Renin–angiotensin system”, “Platelet activation”, and “Complement and coagulation cascades” were significantly enriched, suggesting that acute heat stress may induce gill damage, blood coagulation, and thrombosis in *E. sinensis*, resulting in increased blood pressure and physiological imbalance. Pathways related to phagocytosis such as “Phagosome” and “Fc gamma R-mediated phagocytosis” were activated at the gene level, indicating that phagocytosis plays a key immune function in response to high temperature stress. Then, the activation of “Relaxin signaling pathway” implied that RLN may be essential and responsible for reducing the oxidative damage of gills caused by extreme heat stress. Our findings can provide novel insights into the molecular mechanism of *E. sinensis* under heat stress at transcriptional and translational levels. The mining of key functional genes, proteins and pathways can also provide a basis for the cultivation of new varieties resistant to oxidative stress.

## Figures and Tables

**Table 1:** Real-time quantitative PCR primers used in this study.

Gene Name	Forward Primer Sequence (5′–3′)	Reverse Primer Sequence (5′–3′)
*β-actin*	GGCATCCACGAGACCACTT	CTCCTGCTTGCTGATCCACAT
*PIK3CA*	GCTCAGCATAGGCATAAT	AGGAGATACGCCAAATAG
*CASP7*	GATGCTGGAGGACAGGGAT	TAGCGATGGTTGAAGATG
*HSP70*	CCAGCGTCAAGCAACCAA	TCAACTTCACGGCGGAGC
*HSP90*	AGGTCGTCCGAGTCCACA	GTCGCTGCTCTTCATTCC
*EIF6*	ATCCACGACACCCTACAAG	AGCCCAGTCATTCACTAACA
*ATPase*	CGCCCATCCTCAACTCCT	CTCCACCAAACAGACCAATC
*V-ATPase*	CCAGAACCAGCGTTACCA	CTCCTCTACCCTCAGCATC
*ALF*	TAGTAGTTCCTGGCTGTTCCC	CCTCTATCGGCTGGTTCTG

**Table 2:** DEGs potentially associated with heat shock protein family, antioxidant system, energy metabolism, and immune defense.

Functional Group/Gene	Description	Regulation	MT vs. CT	HT vs. CT	HT vs. MT
Heat shock protein family					
*HSP 70*	Heat shock protein 70	Up	X	X	X
*HSP 90*	Heat shock protein 90	Up	X	X	X
Antioxidant system					
*SOD*	Copper/zinc superoxide dismutase	Up	X	X	X
*GST*	Glutathione S-transferase	Up	X	X	X
*GSH-Px*	Glutathione peroxidase 3	Up			X
Energy metabolism					
*COX*	Cytochrome c oxidase subunit	Up	X		
		Down		X	X
*ND*	NADH dehydrogenase subunit	Up	X		
		Down		X	X
*CYC*	Cytochrome c	Down		X	X
*SDH*	Succinate dehydrogenase	Down		X	X
*IDH*	Isocitrate dehydrogenase	Down		X	
*CS*	Citrate synthase	Down		X	
*ATPase*	ATP synthase	Down		X	X
*V-ATPase*	V-type proton ATPase	Down		X	X
Immune defense					
CTL	LDLa domain-containing C-type lectin	Up	X	X	X
*CYP450*	Cytochrome P450 3A11	Up	X	X	X
*IAP*	Inhibitor of apoptosis protein	Up	X	X	X
Chitinase	Chitinase	Up	X	X	X
*ARE*	Apoptosis-resistant E3 ubiquitin protein	Up	X	X	X
*CASP3*	Caspase 3	Up	X	X	
*CASP7*	Caspase 7	Up	X		
*CASP10*	Caspase 10	Up		X	
*ALF*	Anti-lipopolysaccharide factor	Up		X	X

Note: “X” represents where regulation has occurred.

**Table 3:** Main specific enrichment pathways and differentially expressed genes in the MT vs. CT and HT vs. CT of gills.

Functional Pathway/Gene	Description	Regulation	MT vs. CT	HT vs. CT
Fc gamma R-mediated phagocytosis				
*PTPRC*	Receptor-type tyrosine-protein phosphatase	Up	X	
*PIK3CA*	Phosphoinositide-3 kinase	Up	X	
*RAC1*	Rho-related protein	Up	X	
ECM–receptor interaction				
Integrin α	Integrin alpha	Up	X	
Integrin β	Integrin beta	Up	X	
Regulation of actin cytoskeleton				
Actin	Actin	Up	X	
Integrin α	Integrin alpha	Up	X	
Integrin β	Integrin beta	Up	X	
*PIK3CA*	Phosphoinositide-3 kinase	Up	X	
*RAC1*	Rho-related protein	Up	X	
Toll and Imd signaling pathway				
*Duox*	Dual oxidase	Up	X	
*IAP*	Inhibitor of apoptosis protein	Up	X	
Relaxin signaling pathway				
*RXFP1*	Relaxin family peptide receptor 1	Up		X
*RXFP2*	Relaxin receptor 2	Up		X
*GNAO*	Guanine nucleotide-binding protein G(o) subunit alpha isoform X2	Up		X
*NOS1*	Nitric oxide synthase	Up		X
*PIK3CA*	Phosphoinositide-3 kinase	Up		X
*AC*	Adenylate cyclase	Up		X
Relish	Relish	Up		X
*PKC*	Protein kinase C	Up		X
*PLC*	1-Phosphatidylinositol 4,5-bisphosphate phosphodiesterase	Up		X
*CREB*	cAMP-Responsive element binding protein	Up		X
*VEGF*	Vascular endothelial growth factor	Up		X
*JUN*	Transcription factor AP-1	Up		X
*PKA*	cAMP-Dependent protein kinase catalytic	Up		X
Renin–angiotensin system				
*NAP*	Aminopeptidase N-like	Up		X
*ACE*	Angiotensin-converting enzyme	Up		X

Note: “X” represents where regulation has occurred.

**Table 4:** DEPs potentially associated with heat shock protein family, antioxidant system, and energy metabolism.

Functional Group/Gene	Description	Regulation	MT vs. CT	HT vs. CT	HT vs. MT
Heat shock protein family					
HSP 70	Heat shock protein 70	Up	X	X	X
HSP 60	Heat shock protein 60	Up	X		
Antioxidant system					
CAT	Catalase	Up	X	X	
		Down			X
GST	Glutathione S-transferase	Up	X	X	
		Down			X
Energy metabolism					
COX	Cytochrome c oxidase	Up	X	X	X
ND	NADH dehydrogenase	Up	X	X	
		Down			X
CS	Citrate synthase	Up		X	X
		Down	X		
ATPase	ATP synthase	Up			X

Note: “X” represents where regulation has occurred.

## Data Availability

The data presented in this study are available in the article. Further information is available upon request from the corresponding author.

## Supplementary Materials

The following supporting information can be downloaded at: https://www.mdpi.com/article/10.3390/antiox12122020/s1, Table S1: The accession numbers of qRT-PCR genes in GenBank; Table S2: Statistics of de novo transcriptome assembly; Table S3: Statistical summary of gene functional annotations via BLAST analysis; Table S4: Statistical summary of protein functional annotations; Table S5: The important co-DEG-DEPs at the gene and protein levels; Figure S1: The co-enrichment KEGG pathways “Complement and coagulation cascades” in MT vs. CT (A) and HT vs. CT (B), and “Phagosome” in MT vs. CT (C) and HT vs. CT (D) in transcriptomics; Figure S2: The specific enrichment pathways of MT vs. CT in transcriptomics: “Fc gamma R-mediated phagocytosis” (A), “Regulation of actin cytoskeleton” (B), and “Toll and Imd signaling pathway” (C), and the specific enrichment pathways of HT vs. CT “Relaxin signaling pathway” (D) and “Renin–angiotensin system” (E); Figure S3: The top two enriched pathways in HT vs. MT in transcriptomics: “Oxidative phosphorylation” (A) and “Thermogenesis” (B); Figure S4: The enrichment pathways of MT vs. CT in proteomics: “Lysosome” (A) and “Platelet activation” (B); Figure S5: The enrichment pathways of MT vs. CT in proteomics: “Apoptosis” (A), “Relaxin signaling pathway” (B), and “Phospholipase D signaling pathway” (C); Figure S6: The enrichment pathways “Platelet activation” of MT vs. CT in transcriptomics (A) and HT vs. MT in proteomics (B).
