# Supplementary material for: Integration of Transcriptomics and Proteomics Analysis Reveals the Molecular Mechanism of Eriocheir sinensis Gills Exposed to Heat Stress
Source: Antioxidants (Basel). 2023 Nov 21;12(12):2020. doi: 10.3390/antiox12122020 (PMC10740794; doi:10.3390/antiox12122020)
Supplement: Supplementary file 1 [file antioxidants-12-02020-s001.zip › Table S1.pdf]

Table S1. The accession numbers of qRT-PCR genes in GenBank.

Gene name	Accession number
β-actin	OR813940
PIK3CA	OR813941
CASP7	OR813942
HSP70	OR813943
HSP90	OR813944
EIF6	OR813945
ATPase	OR813946
V-ATPase	OR813947
ALF	OR813948
