# Supplementary material for: Integration of Transcriptomics and Proteomics Analysis Reveals the Molecular Mechanism of Eriocheir sinensis Gills Exposed to Heat Stress
Source: Antioxidants (Basel). 2023 Nov 21;12(12):2020. doi: 10.3390/antiox12122020 (PMC10740794; doi:10.3390/antiox12122020)
Supplement: Supplementary file 1 [file antioxidants-12-02020-s001.zip › Table S2.pdf]

Table S2. Statistics of de novo transcriptome assembly.

Type	Transcripts	Unigenes
Total sequence num	77,708	49,754
Total sequence base	73,559,604	45,810,342
Percent GC	46.22	46.47
Largest length	20,040	20,040
Smallest length	201	201
Average length	946.62	920.74
N50	1,662	1,740
