# Supplementary material for: Integration of Transcriptomics and Proteomics Analysis Reveals the Molecular Mechanism of Eriocheir sinensis Gills Exposed to Heat Stress
Source: Antioxidants (Basel). 2023 Nov 21;12(12):2020. doi: 10.3390/antiox12122020 (PMC10740794; doi:10.3390/antiox12122020)
Supplement: Supplementary file 1 [file antioxidants-12-02020-s001.zip › Table S3.pdf]

Table S3. Statistical summary of gene functional annotations by BLAST analysis.

Database	Number of annotated unigenes	Percentage of annoted unigenes (%)
Nr	17,960	36.10
Pfam	12,032	22.55
Swiss-prot	11,218	26.30
COG	13,083	24.18
GO	11,070	22.25
KEGG	10,947	22.00
