# Supplementary material for: Integration of Transcriptomics and Proteomics Analysis Reveals the Molecular Mechanism of Eriocheir sinensis Gills Exposed to Heat Stress
Source: Antioxidants (Basel). 2023 Nov 21;12(12):2020. doi: 10.3390/antiox12122020 (PMC10740794; doi:10.3390/antiox12122020)
Supplement: Supplementary file 1 [file antioxidants-12-02020-s001.zip › Table S4.pdf]

Table S4. Statistical summary of protein functional annotations.

Database	Number of annotated protein	Percentage of annoted protein (%)
COG	490	95.33
GO	424	82.49
KEGG	420	81.71
Pfam	490	95.33
SubCell-Location	514	1.00
Total	514	1.00
