# Supplementary material for: Integration of Transcriptomics and Proteomics Analysis Reveals the Molecular Mechanism of Eriocheir sinensis Gills Exposed to Heat Stress
Source: Antioxidants (Basel). 2023 Nov 21;12(12):2020. doi: 10.3390/antiox12122020 (PMC10740794; doi:10.3390/antiox12122020)
Supplement: Supplementary file 1 [file antioxidants-12-02020-s001.zip › Table S5.pdf]

Table S5 The important co-DEG-DEPs at the gene and protein levels.

Treatment	ID	Gene	Annotation	Transcriptomics		Proteomics	
				Log2FC	Regulation	Log2FC	Regulation
MT vs CT	TRINITY_DN2251_c0_g2_i1_orfp1	*Chitinase 1*	Chitinase 1 [*Eriocheir sinensis*]	2.72	Up	1.20	Up
	TRINITY_DN1273_c0_g1_i1_orfp1	*GST*	Glutathione S-transferase [*Eriocheir sinensis*]	1.62	Up	1.50	Up
	TRINITY_DN9094_c0_g1_i1_orfp1	*Integrin β3*	Integrin beta 3 [*Eriocheir sinensis*]	1.28	Up	1.60	Up
	TRINITY_DN5613_c0_g1_i6_orfp1	*PHC*	Pseudohemocyanin-2-like [*Artibeus jamaicensis*]	3.39	Up	1.20	Up
	TRINITY_DN11232_c0_g1_i1_orfp1	*HSP70*	Heat shock protein 70 [*Eriocheir sinensis*]	2.49	Up	6.42	Up
HT vs CT	TRINITY_DN3182_c0_g1_i3_orfp1	*HC*	hemocyanin subunit 3 [*Portunus pelagicus*]	4.26	Up	1.50	Up
	TRINITY_DN5534_c0_g1_i2_orfp1	*Ficolin-1*	Ficolin-1 [*Chionoecetes opilio*]	3.98	Up	5.94	Up
	TRINITY_DN814_c0_g2_i9_orfp1	*RDH12*	Retinol dehydrogenase 12 [*Chionoecetes opilio*]	2.46	Up	3.50	Up
