# Supplementary figures and images for: Integration of Transcriptomics and Proteomics Analysis Reveals the Molecular Mechanism of Eriocheir sinensis Gills Exposed to Heat Stress
Source: Antioxidants (Basel). 2023 Nov 21;12(12):2020. doi: 10.3390/antiox12122020 (PMC10740794; doi:10.3390/antiox12122020)

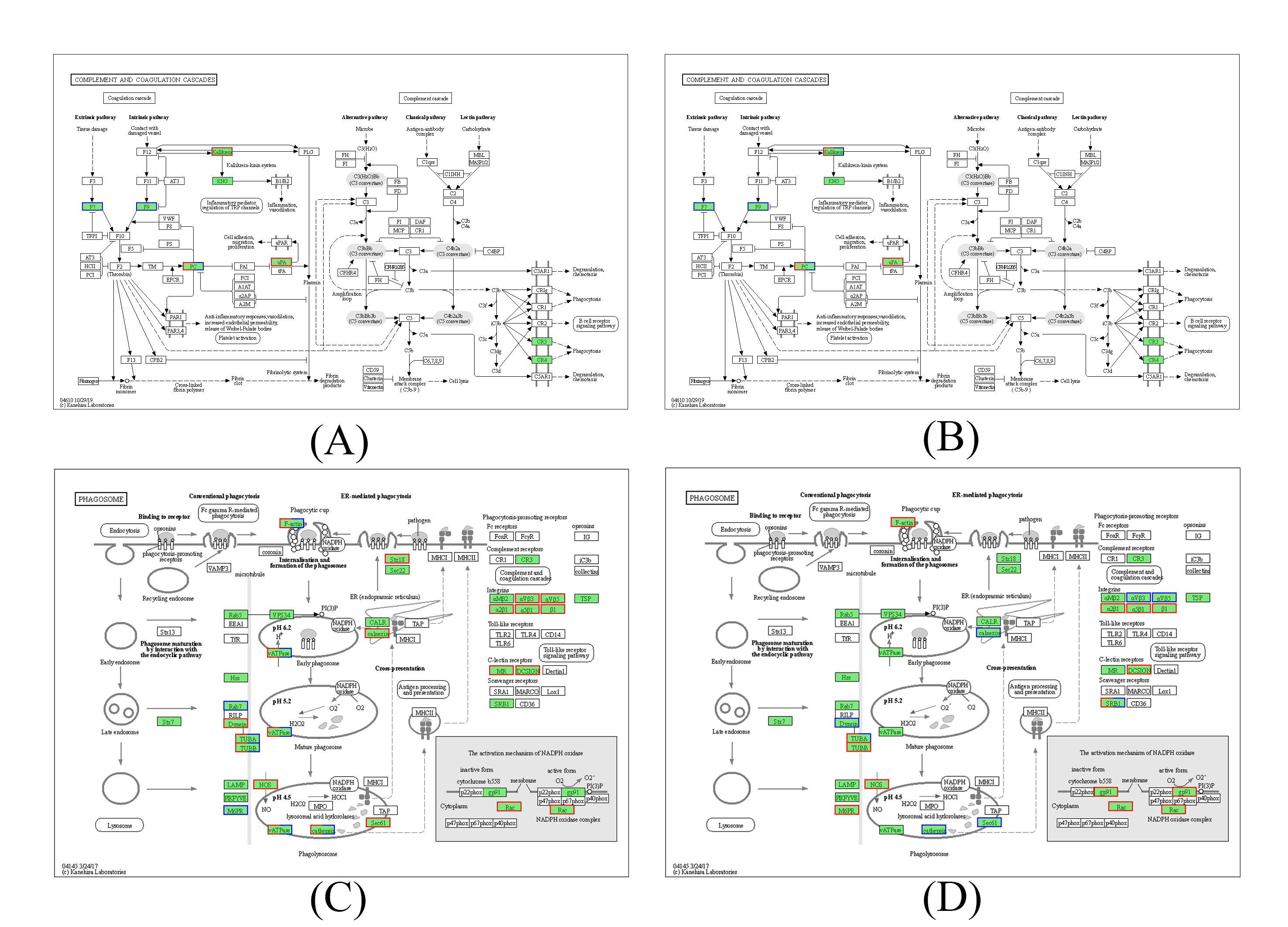

Supplement: Supplementary file 1 [file antioxidants-12-02020-s001.zip › Fig. S1.tif]

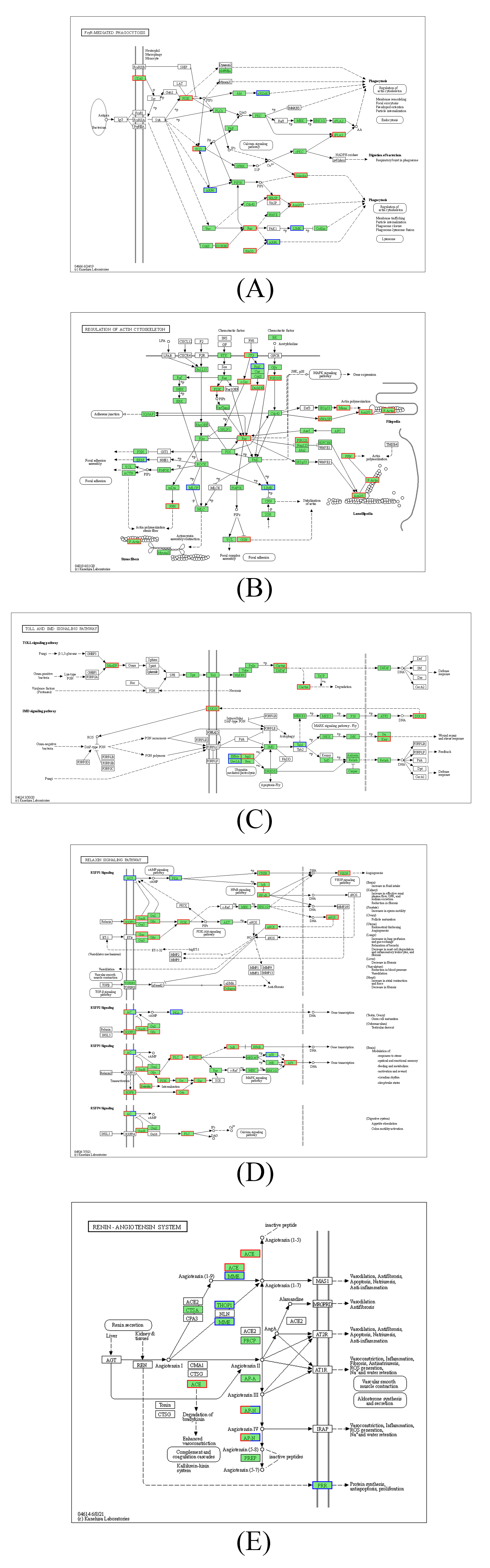

Supplement: Supplementary file 1 [file antioxidants-12-02020-s001.zip › Fig. S2.tif]

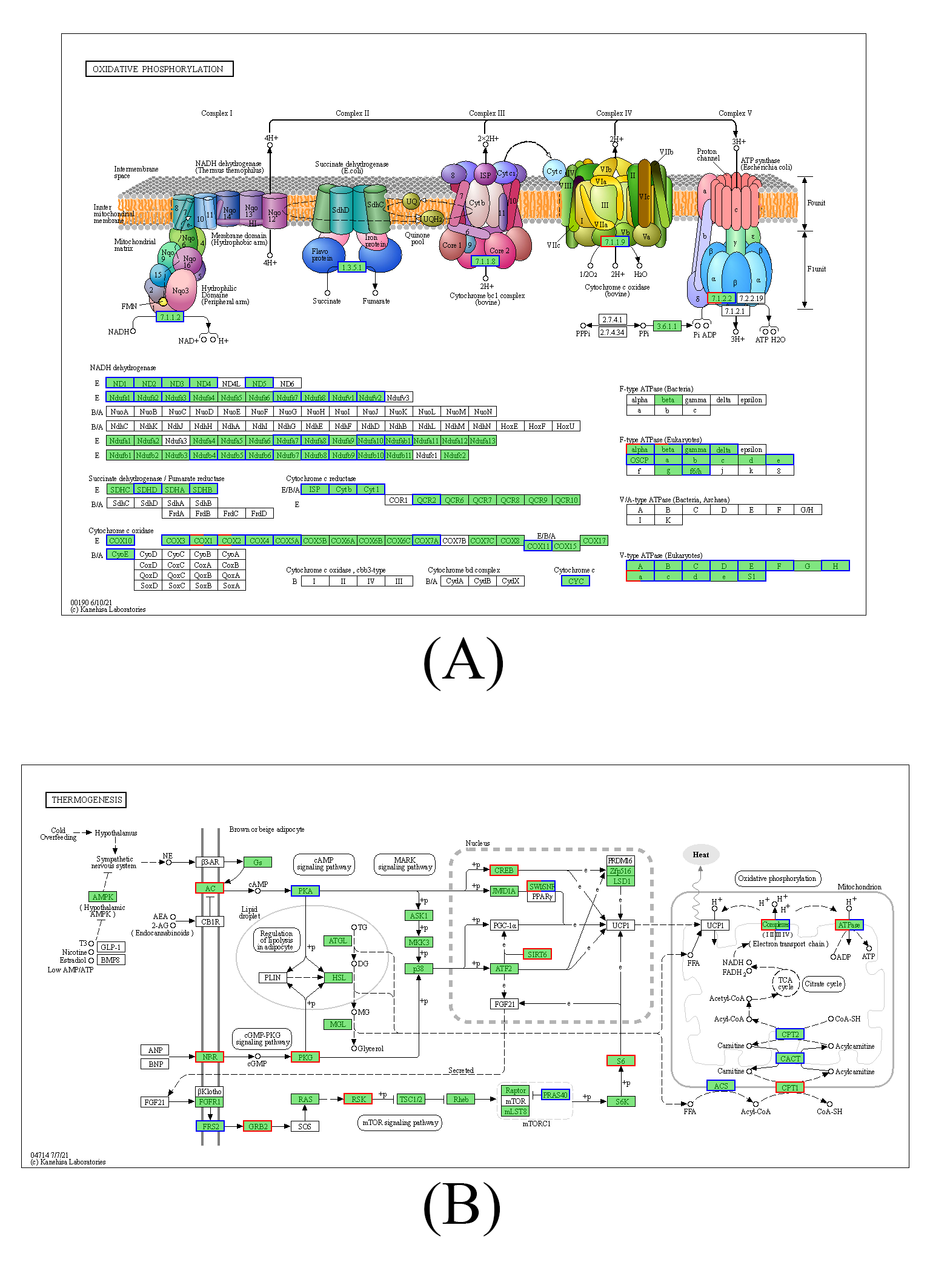

Supplement: Supplementary file 1 [file antioxidants-12-02020-s001.zip › Fig. S3.tif]

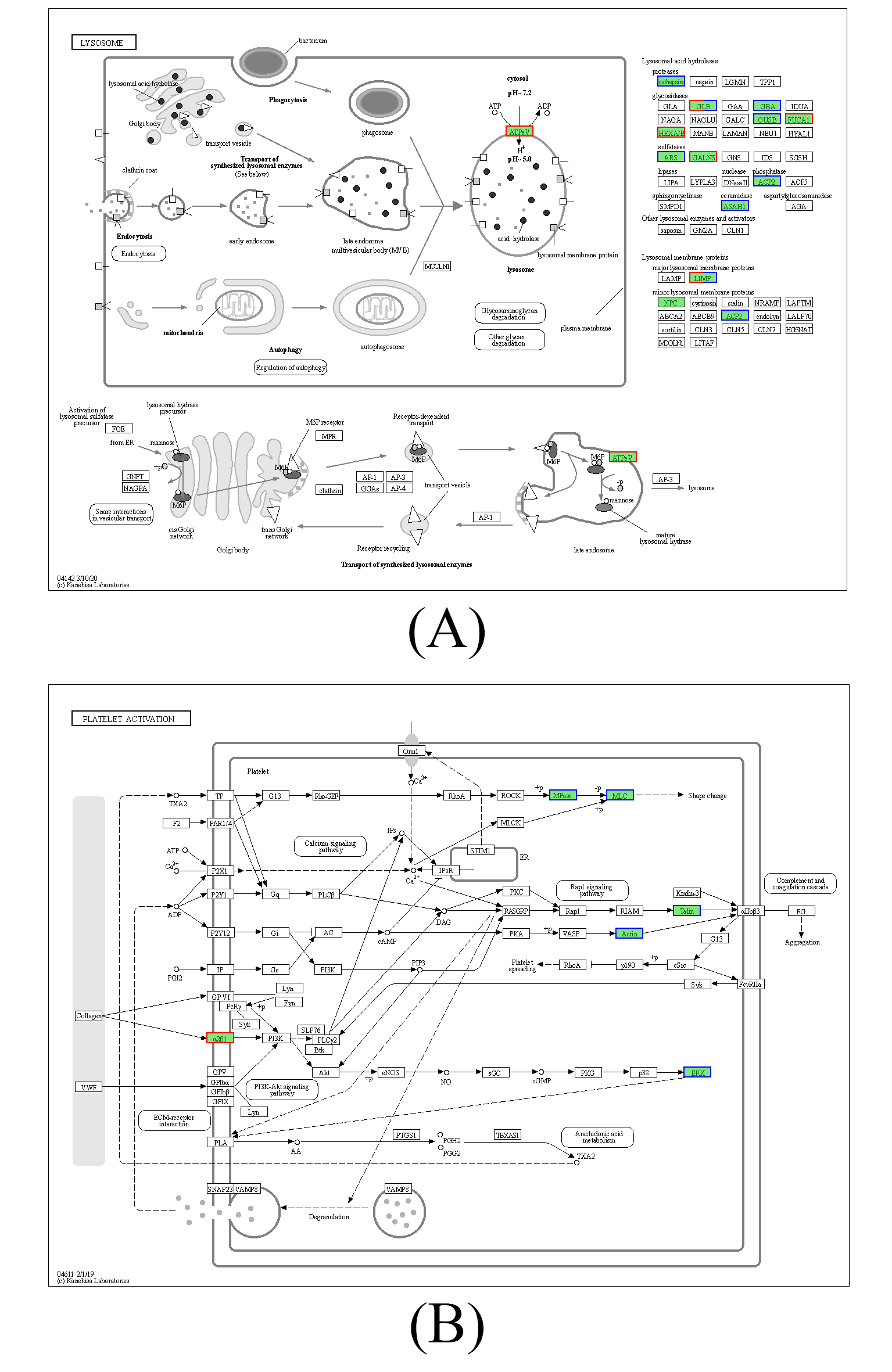

Supplement: Supplementary file 1 [file antioxidants-12-02020-s001.zip › Fig. S4.tif]

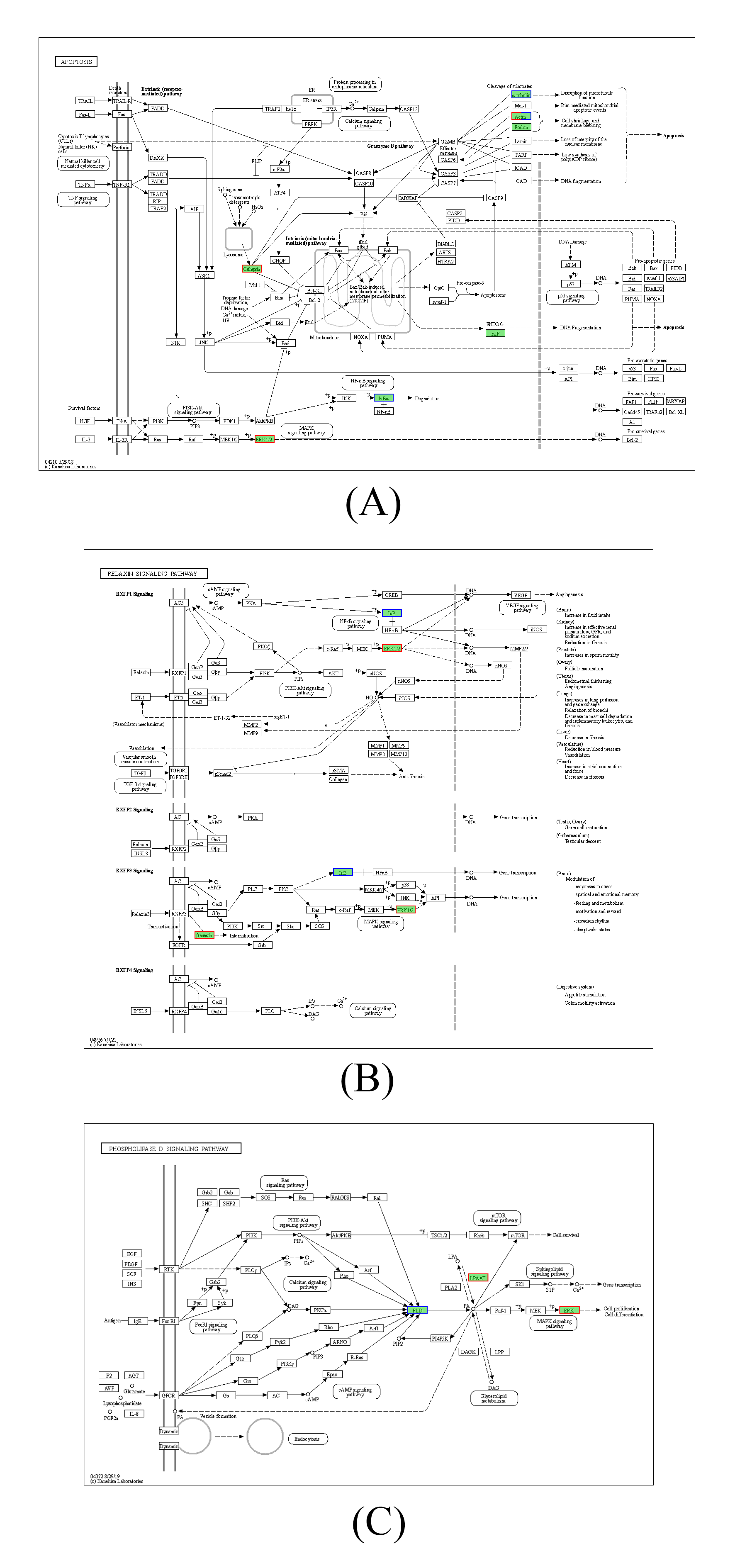

Supplement: Supplementary file 1 [file antioxidants-12-02020-s001.zip › Fig. S5.tif]

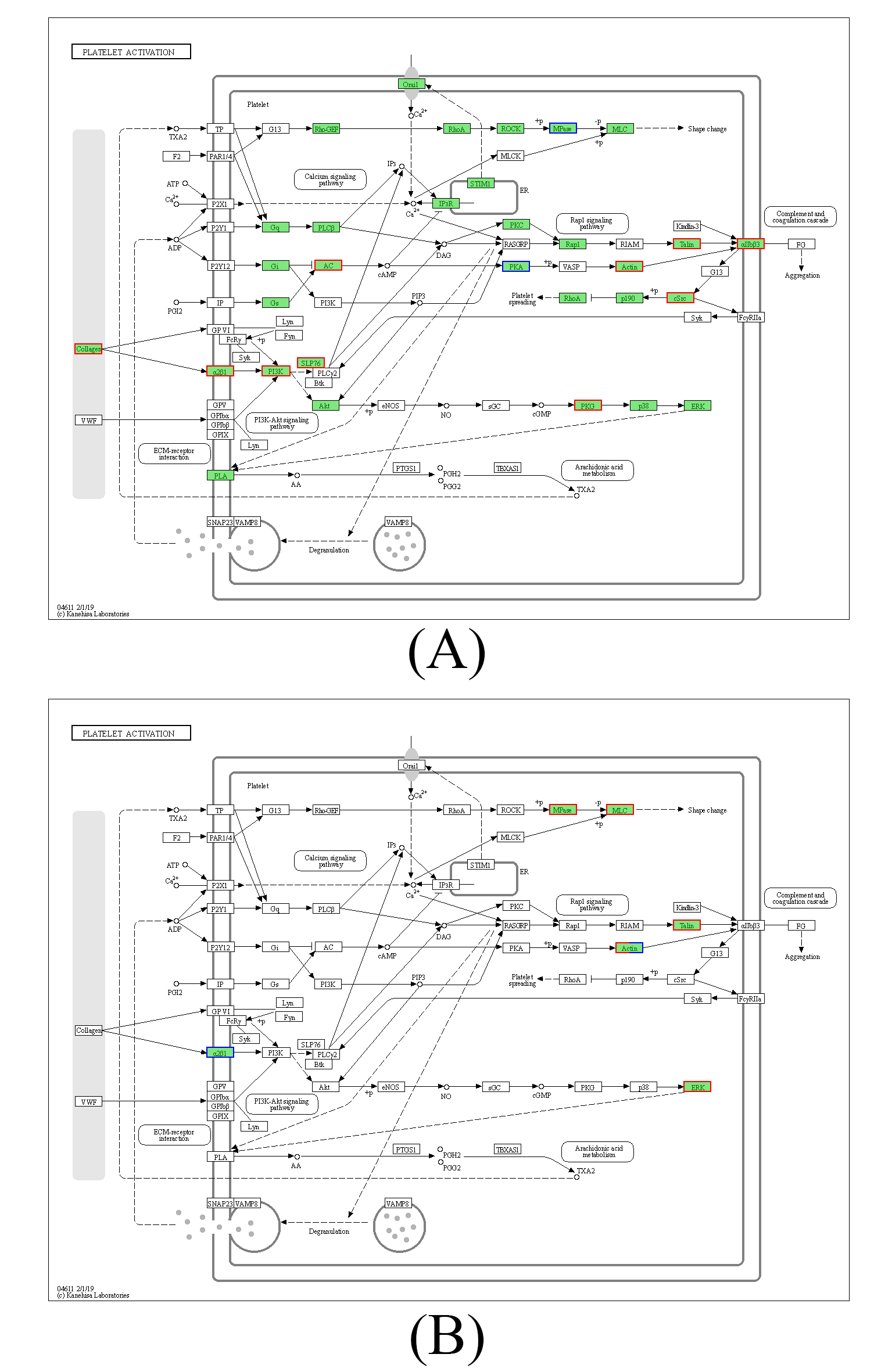

Supplement: Supplementary file 1 [file antioxidants-12-02020-s001.zip › Fig. S6.tif]
